# Chemical Composition, Antioxidant and Antimicrobial Activity of Essential Oils from Organic Fennel, Parsley, and Lavender from Spain

**DOI:** 10.3390/foods5010018

**Published:** 2016-03-04

**Authors:** Irene Marín, Estrella Sayas-Barberá, Manuel Viuda-Martos, Casilda Navarro, Esther Sendra

**Affiliations:** IPOA Research Group, Agro-food Technology Department, School of Engineering of Orihuela, Miguel Hernandez University, Ctra. De Beniel km 3.2. 03312 Orihuela, Alicante, Spain; irene_marin_84@hotmail.com (I.M.); estrella.sayas@umh.es (E.S.-B.); mviuda@umh.es (M.V.-M.); casilda.navarro@umh.es (C.N.)

**Keywords:** antioxidant, antimicrobial, organics, fennel, parsley, lavender

## Abstract

The aim of this work was to (i) determine the chemical composition of the essential oils of three spices widely cultivated in Spain from organic growth: *Foeniculum vulgare*, *Petroselium crispum*, and *Lavandula officinalis*; (ii) determine the total phenolic content; (iii) determine the antioxidant activity of the essentials oils by means of three different antioxidant tests and (iv) determine the effectiveness of these essentials oils on the inhibition of *Listeria innocua* CECT 910 and *Pseudomonas fluorescens* CECT 844. There is a great variability in the chemical composition of the essential oils. Parsley had the highest phenolic content. Overall, parsley presented the best antioxidant profile, given its highest % of inhibition of DPPH radical (64.28%) and FRAP (0.93 mmol/L Trolox), but had a pro-oxidative behavior by TBARS. Lavender essential oil showed the highest antibacterial activity against *L. innocua* (>13 mm of inhibition at 20–40 μL oil in the discs), followed by parsley with an inhibition zone of 10 mm (when more than 5 μL oil in the discs), and fennel 10 mm (when more than 40 μL oil in the discs). *P. fluorescens* was not inhibited by the tested essential oils.

## 1. Introduction

Oxidation is one of the most common spoilage mechanisms of foods; in fact there is a widespread use of antioxidants in foods. Some of them are obtained by chemical synthesis, and as consumers prefer natural products, there is a growing demand for natural sources of antioxidants. Several plant essential oils (EOs) have a long history of being used in foods, and are considered GRAS, when such oils are obtained from organic grown plants by approved procedures they can also be incorporated into organic foods. The European Commission [1] has strategic plans to promote organic farming in the European Union. Nowadays, EOs are mainly used in the food industry as flavoring agents, and are used as well by the hygienic, pharmaceutical, cosmetic, and perfume industries [2].

The food industry also benefits from EOs preservative properties [2,3]. Meat products, soups, dairy products (cheeses, creams), flavored oils and vinegars, and fermented vegetables, among others, usually contain EOs or other plant parts or extracts. In order to enhance food safety it is of major interest to investigate the antimicrobial properties of EOs, especially on food spoilage and pathogenic microorganisms, as well as the interactions among food-EOs-microorganisms and possible combinations of antimicrobial agents.

Limiting factors of the use of EOs in foods as preserving agents are (i) EOs are potent flavoring agents and are acceptable, from a sensory point of view, for specific foods; (ii) EOs addition into foods is common at reduced concentrations, sometimes below effective antimicrobial concentrations; (iii) they are not usually effective antimicrobials by themselves and rather need combination with other antimicrobial agents.

Most antimicrobial studies of EOs have been carried out on bacteria, and to a lesser extent on molds and yeasts. As a common trend, gram-negative bacteria have lower susceptibility to EOs than gram-positive ones [4], mainly due to their membrane characteristics that act as barriers against macromolecules and hydrophobic compounds. Given that EOs are hydrophobic compounds, gram negative bacteria are somehow protected against them [5].

Antioxidant properties of EOs have also been reported. Antioxidant compounds pose the ability to delay or inhibit the oxidation of lipids and other molecules by inhibiting the initiation or propagation of oxidation chain reactions [6]. The association of the myriad of compounds present in EOs provides higher antioxidant activity than the summed activity of the individual components [7,8,9,10,11]. According to Zeng and Wang [12], EOs may be used as food preserving agents mainly due to the presence of phenolic compounds as main components, which are responsible for the antioxidant properties and may be an alternative to the use of synthetic antioxidants.

Given the potential of EOs as antimicrobials and antioxidants, it is of great interest to study *in vitro* properties of organic EOs. Their knowledge may allow their proper use in organic foods, and also pose an alternative to synthetic antioxidants for conventionally produced foods. The present study is focused in three EOs from widely used species of mild taste and flavor: fennel (*Foeniculum vulgare*), parsley (*Petroselium crispum*), and lavender (*Lavandula officinalis*), obtained from organic grown plants cultivated in Spain. EOs composition, phenolic content, antioxidant properties, and antimicrobial properties against two psycrotrophic bacteria, one responsible of food spoilage (*Pseudomonas fluorescens* CECT *844*) and the other indicator of the presence of *Listeria* spp. (*Listeria innocua* CECT *910*) are investigated.

## 2. Experimental Section

*Chemicals*: Ascorbic acid, butylated hydroxytoluene (BHT), 2,2′-diphenyl-1-picrylhydrazyl (DPPH), ferrozine, iron(III) chloride, iron(II) chloride, trichloroacetic acid (TCA), pentane, and Trolox were from Sigma Chemical Company (Germany). Potassium hydrogen phosphate, anhydrous sodium sulphate, 2-thiobarbituric acid (TBA), and disodium hydrogen phosphate were from Merck (Darmstadt, Germany). Potassium ferricyanide was from Fluka BioChemika (Neu Ulm, Germany). The solvent used for preparing standard solutions was methanol of HPLC grade, supplied by Merck. Solutions were freshly prepared, all flasks and vials were of amber glass and were kept in darkness.

*Plant materials*: Fennel (*Foeniculum vulgare*), parsley (*Petroselium crispum*), and lavender (*Lavandula officinalis*) commercial essential oils from organic grown plants were purchased from Herbes del Molí (Benimarfull, Alicante, Spain). The plantation and company are certified for organic agriculture by CAECV (Comité de Agricultura Ecológica de la Comunitat Valenciana). EOs were extracted from fennel plants, parsley plants, and lavender flowers and plants by hydrodistillation. The company reported an extraction yield for lavender of 3.45 mL/100 g dry weight, no yield data was recorded by the company for fennel and parsley EOs.

*GC-MS and GC-FID Analytical Conditions*: The volatile compounds were isolated, identified and quantified as described in a previous work [13]. A Shimadzu GC-17A gas chromatograph (Shimadzu Corporation, Kyoto, Japan), coupled with a Shimadzu mass spectrometer detector (GC-MS QP-5050A, Shimadzu, Kyoto, Japan) was used for peaks identification. The GC-MS system was equipped with a TRACSIL Meta X5 column (Teknokroma S. Coop. C. Ltd, Barcelona, Spain; 30 m × 0.25 mm i.d., 0.25 μm film thickness). Analyses were carried out using helium as carrier gas at a flow rate of 1.0 mL/min, at a split ratio of 1:10 and the following temperature programme: 40 °C for 5 min; rising at 3.0 °C/min to 200 °C and held for 1 min; rising at 15 °C/min to 280 °C and held for 10 min. Injector and detector were held at 250 °C and 300 °C, respectively. Diluted samples (1/10 pentane, *v/v*) of 0.2 μL of the extracts were always injected. Mass spectra were obtained by electron ionization (EI) at 70 eV, using a spectral range of 45–450 *m/z*. Most of the compounds were identified by simultaneously using two different analytical methods [14]: (a) KI, Kováts Index in reference to n-alkanes (C_8_-C_32_); and (b) mass spectra (authentic chemicals and Wiley spectral library collection). Identification was considered tentative when it was based on mass spectral data only. Semi-quantification of compounds was run in a Shimadzu GC-2100 equipped with an FID detector and the same column previously described and the same flow and oven conditions. 0.2 μL were injected manually in the split mode (split ratio 1/44). Quantitative data were obtained electronically from FID area data without using correction factors. All the tests were performed in triplicate.

*Total phenolic content*: Total Phenolic Content was assessed by Folin-Cicalteau method [15].

*Antioxidant activity:* (1) *2*,*2*′*-diphenyl-1-picrylhydrazyl (DPPH) radical scavenging method:* The antioxidant activity of fennel, parsley, and lavender essential oils was measured in terms of hydrogen donating or radical scavenging ability, using the stable radical DPPH [16]. The amount of sample necessary to decrease the absorbance of DPPH (IC_50_) by 50% was calculated graphically. Each assay was carried out in triplicate; (2) *Ferric reducing antioxidant power**:*** The ferric reducing power (FRAP) of the essential oils was determined by using the potassium ferricyanide-ferric chloride method [16]. The FRAP of a sample is estimated in terms of Trolox equivalent antioxidant capacity (TEAC) in mmol/L Trolox. Each assay was carried out in triplicate; (3) *Inhibition of lipid peroxidation of buffered egg yolk by essential oils:* The method of Daker *et al.* [17] was modified, to determine the thiobarbituric acid reactive substance (TBARS), a secondary product of lipid peroxidation. Each assay was carried out in triplicate.

*Microbial strains:* The essentials oils were individually tested against *Listeria innocua* CECT 910 and *Pseudomonas fluorescens* CECT 844 from the Spanish Type Culture Collection (CECT) of the University of Valencia.

*Agar disc diffusion method:* The agar disc diffusion method described by Tepe *et al.* [18] with some modifications used to determine the antibacterial capacity of the essential oils. Briefly, a suspension (0.1 mL of 10^6^ CFU mL^−1^) of *Listeria innocua* was spread on the solid medium plates (BHI agar; Sharlab, Sharlab SL, Barcelona, Spain). Sterile filter paper discs, 9 mm in diameter (Schlinder & Schuell, Dassel, Germany) were impregnated with 40 μL of the oil and placed on the inoculated plates; these plates were incubated at 37 °C for 24 h. *Pseudomonas fluorescens*, was cultured in nutritive agar II (Oxoid, Basingstoke, Hampshire, England), and incubated at 26 °C for 48 h. The diameters of the inhibition zones were measured in millimeters. All tests were performed in triplicate.

*Determination of volume effect:* The volume effect (VE) was studied to ascertain which amounts of essential oil had an inhibitory effect on bacterial growth in the disc diffusion assay. The culture techniques used were those described in the previous paragraph (Agar disc diffusion method), but adding 40, 20, 10, 5, and 2.5 μL of essential oil [19]. All tests were performed in triplicate.

*Statistical analysis*: Data on antioxidant and antibacterial activities were analyzed by means of multivariate procedure GLM (General Lineal Model). For comparison among means Tukey’s test was used (*p* < 0.05) [20]. Antioxidant activity was studied by means of ANOVA test with two factors (EO type: fennel, parsley, and lavender and concentration: 20, 15, 10, and 5 g/L). For antibacterial activity one-way ANOVA for each EO was applied being the factor oil volume in de discs (40, 20, 10, 5, and 2 μL). All determinations were run on SPSS^®^ IBM^®^ Statistics 23.0.0.0. software (International Business Machines Corp., Armonk, New York, USA).

## 3. Results and Discussion

### 3.1. Essential Oils Chemical Composition

Table 1 presents EO’s composition as analyzed by GC-MS and CG-FID and identified by Wiley Library and Kòvats index. Seventeen compounds were identified in organic fennel EO; major component was limonene (26.44%) followed by anethole (23.5%) and fenchone (21.68%), and finally α-phellandrene and α-pinene (9.26% and 6.22%, respectively) adding up to 87.1% of total EO composition. Viuda-Martos *et al.* [13] in Egyptian organic fennel reported *trans*-anethole (65.59%) as major component, followed by methyl-chavicol (13.11%). Limonene and fenchone were also present as in Spanish organic fennel (8.54% and 7.76%, respectively). Telci *et al.* [21] in Turkish fennel also reported *trans*-anethole as major component of the EO and similar content of methyl-chavicol, limonene and fenchone. Politeo *et al.* [22] studied the chemical composition of 12 EOs from Croatia, reported main components of fennel EO being: 77.6% *trans*-anethole (in agreement with Viuda-Martos *et al.* [13] and Telci *et al.* [21]), followed by 12.4% of fenchone. Cerpa Chávez [23] reported that *trans*-anethole was the main component, followed by fenchone, α-pinene, methyl-chavicol, α-phellandrene, and d-limonene. Given this scenario, it can be assumed that the fennel chemo type used in the present study differed from the others and does not belong to the *trans*-anethole chemo type [24], according to Raal *et al.* [25] who studied the composition of fennel EO from several European countries, *trans*-anethole content was always the main component and ranged from 34.8% to 82.0% in the EO. Napoli *et al.* [26] reported an only wild variety of fennel from Sicily were *trans*-anethole was not the main component, and limonene accounted for a 34% of the EO. Many of the referred cites report results from EOs obtained from fennel fruit and seeds; whereas the present results are referred to fennel plant which may explain some of the differences.

Regarding organic parsley, 24 compounds were identified. Main components were myristicin (36.15%), apiole (20.97%), α-pinene (15.47%), and β-pinene (10.43%). The presence of ally-tetramethoxy-bencene, limonene, and elemicin (6.45%, 4.74%, and 2.74%, respectively) was also relevant. Similar profiles were reported by Zhang *et al.* [28] (major component myristicin (32.75%), apiole (17.54%), α-pinene (16.64%), and β-pinene (11.54%)); whereas Viuda-Martos *et al.* [13] in Egyptian organic parsley reported that main components were apiole (46.46%) followed by α-pinene (22.21%), and β-pinene (16.06%), so it can be assumed that it was from a different parsley cultivar.

A total of 36 compounds were identified in Spanish organic lavender EO. Major components were linalool (34.44%) followed by linalyl acetate (34.19%) accounting up to 69% of the total composition. Other relevant components were *trans-*β-o-cymene (5.05%), lavandulyl acetate (4.08%), β-cariophyllene (3.83%), *trans*-linalool-oxide (3.44%), and β-farnesene (3.08%). Viuda-Martos *et al.* [13] reported similar main components in Egyptian organic lavender with slight differences in proportions (39.83% linalool and 32.11% linalyl acetate), however minor compounds differed substantially: camphor (11.29%) and β-phellandrene (7.63%). Hassiotis *et al.* [29] reported in Greek lavender the same major components: linalool (26.9%) and linalyl acetate (22.8%).

### 3.2. Total Phenolic Content

Phenolic compounds are secondary metabolites present in whole plants; they share in common an aromatic ring and one or several substitutions with hydroxyl groups. They can be classified into phenols, phenolic acids, phenyl-propanoids, flavonoids, tannins and quinones, or alcohols like those present in EOs [30,31], many of these compounds have proven antioxidant properties [32]. In Table 2, total phenolic content (TPC) of organic EOs of fennel, parsley, and lavender are presented. Parsley presented the highest TPC followed by fennel and lavender. Viuda-Martos *et al.* [13] reported similar TPC in parsley and lavender from Egypt. Several other studies reported TPC in fennel from around the world, most of them with lower values than the reported in the present study [13,33,34,35,36], which may be related to the different fennel chemo type, or plant part, as stated previously.

### 3.3. Antioxidant Activity of the Essential Oils

It is well known that antioxidant mechanisms are complex and diverse, and so antioxidant properties need to be assessed by several methods in order to better understand and determine the antioxidant ability [19].

#### 3.3.1. Inhibition of DPPH Radical

The antioxidant activity of organic fennel, parsley, and lavender EOs was assessed by evaluating hydrogen donating ability, or radical scavenging activity, using the stable radical DPPH. As hydrogen is donated to DPPH the color fades. The higher the ability to donate hydrogen, the more intense the bleaching effect, and so the lower will be the IC_50_ [37]. Values for DPPH radical scavenging ability of the EOs is presented in Table 3. Organic parsley EO was the most effective radical scavenger (*p* < 0.05) at all tested concentrations, followed by organic lavender, whose effect was significantly different depending on the concentration tested. Fennel showed the lowest radical scavenging ability at all tested concentrations. The present results are similar to those reported by Viuda-Martos *et al.* [13] for Egyptian organic fennel, parsley, and lavender. Regarding IC_50_ (EO concentration to inhibit 50% of the radicals) the order was as follows: BHT > parsley EO > lavender EO > fennel EO.

Several authors have reported a lineal relation among TPC and antioxidant properties [38,39] in the present study such relation was confirmed as increased concentration of the same essential oil yield higher inhibition of DPPH radical. However, when comparing among EOs such a relation is not obvious, fennel again being the “outlier”. In fact, not only TPC but also phenolic profile may lead to different antioxidant properties [40], as an example, DPPH radical scavenging ability of (+)-catechin is not as good as of quercetin [39]. Also, essential oil components greatly differ on antioxidant ability, with few exceptions monoterpene hydrocarbons are more effective antioxidants than sesquiterpenes and non-isoprenoid components [7].

#### 3.3.2. Ferric Reducing Antioxidant Activity of Essential Oils

Ferric reducing capacity method is based on the ability of EOs to reduce the complex ferric/ferricianin to ferrous form [41]. In Table 4, ferric reducing ability of organic fennel, parsley, and lavender EOs is expressed in terms of Trolox concentration. All tested essential oils showed some antioxidant activity by this mechanism, such activity was linearly dependent on EO concentration (except for lavender at 20 and 50 g/L were no significant differences were detected). For FRAP results there was a lineal relation among TPC and antioxidant activity, so parsley EO showed the highest antioxidant activity (*p* < 0.05) followed by fennel and lavender. Similar results were reported by Viuda-Martos *et al.* [13] for Egyptian organic EOs. Martucci *et al.* [42] used lavender EO in edible films and reported FRAP values higher than the reported in the present study.

#### 3.3.3. Inhibition of Lipid Peroxidation of Buffered Egg Yolk by Essential Oils (TBARS)

From the tested methods, TBARS is considered the one that best approaches real antioxidant behavior in foods. TBARS test is used to determine secondary metabolites from oxidation reactions (malonaldehyde) of lipids [17]. Malonaldehyde reacts with TBA and yields a pink color in the solution that can be measured by a spectrophotometer; EO is added to inhibit the reaction, and in the present test the EO is added to inhibit the oxidation of egg yolk phospholipids as compared to the antioxidant ability of BHT. In Table 5, TBARS results for the tested EOs are presented. Organic fennel and lavender inhibited TBARS formation but organic parsley had pro-oxidant activity at concentrations over 5 g/L. Inhibitory activity of TBARS was not linearly correlated to oil concentration. Viuda-Martos *et al.* [13] on Egyptian organic EO’s reported inhibition of TBARS linearly correlated with EOs concentration and did not report pro-oxidant activity of parsley EO. Given differences among parsley composition of both studies, the higher content of sesquiterpenes in Egyptian parsley may be the cause of the higher antioxidant activity of such oil. TPC of EOs did not linearly correlate with their ability to inhibit TBARS, as occurred with DPPH test. Table 6 presents a summary of the TPC and antioxidant ability of the tested organic EOs, antioxidant activity of the tested organic EOs followed different mechanisms as each oil behaved differently depending on the method of evaluation, supporting the fact that an antioxidant only method does not fully reflect the antioxidant ability of a substance, as well as the need to use the studied oils in combination with other EOs or substances in order to achieve a wide antioxidant spectrum.

### 3.4. Antimicrobial Activity of Organic EOs

Disc diffusion method was used to determine the inhibitory effect of Spanish organic EOs against *L. innocua* CECT 910 and *P. fluorescens* CECT 844*.* Table 7 presents the inhibitory halus of the EOs at the tested oil concentrations. All tested oils presented a moderate to low activity against *L. innocua* being the most effective organic lavender (when 20–40 μL were present), followed by parsley (when from 5 to 40 μL were present, not in a concentration dependent manner) and finally fennel that was the least effective. None of the studied EOs was effective in inhibiting *P. fluorescens*, which is a gram negative bacterium, which generally are less sensitive to EOs than gram positive ones. Oussalah *et al.* [3] and Viuda-Martos *et al.* [13] suggested that such differences among gram positive and negative are due to different membranes/cell walls and subsequent differences in the ability to get disturbed by the oils, modify enzymatic systems, plasma coagulation, DNA damage, or alteration of membrane proteins, that may allow altered permeability. Similar antibacterial behavior has been reported for Egyptian organic lavender, parsley, and fennel EOs [13] for both *L. innocua* and *P. fluorescens*.

The mechanisms by which EOs exert antimicrobial effect are not fully known; oil chemical composition, synergy among oil components, even minor components may be the responsible [43]. As seen in Table 1, the studied oils do not contain relevant amounts of compounds of proven antimicrobial effect. Fennel, parsley, and lavender are not effective antimicrobials for the tested bacteria so they need to be added together with other organic EOs or substances in order to provide effective preserving effect.

## 4. Conclusions

The study of the antioxidant activity of EOs from organic fennel (*Foenicum vulgare*), parsley (*Petroselium crispum*), and lavender (*Lavandula officinalis*) points to a moderate to low antioxidant activity of the oils and, what is more important, to different mechanisms of antioxidant action for each oil and so to the need of combining these oils with other antioxidant substances in order to achieve a proper antioxidant spectrum. The evaluated organic parsley EO (main components: myristicin and apiole) is even pro-oxidant by TBARS method. The evaluated organic fennel is from an uncommon chemo type as *trans*-anethole is not the main component.

Regarding antimicrobial activity, all tested oils moderately inhibit *L. innocua* (gram positive), lavender being the most effective, however none of them are able to inhibit *P. fluorescens*. Further studies need to focus on the study of possible synergies with other organic oils or natural substances in order to achieve preserving properties with GRAS substances to be used for organic foods.

## Figures and Tables

**Table 1:** Principal constituents of organic lavender, fennel, and parsley essential oils from Spain and their relative percentages of total chromatogram area, and Kovats Index.

Composition	Id. ^1^	Kováts Indexes		*Lavandula officinalis* (%)	*Foeniculum vulgare* (%)	*Petroselinum crispum* (%)
		KI	Lit ^2^			
α-thujene	KI,W	920	923	0.07	0.14	0.15
α-pinene	KI,W	927	933	0.26	6.22	15.47
Camphene	KI,W	939	952	0.25	0.59	0.10
Sabinene	KI,W	966	973	-	0.14	-
β-pinene	KI,W	978	980	0.05	2.04	10.43
Octen-3-one	KI,W	980	976	1.35	-	-
Myrcene	KI,W	985	991	0.62	2.27	0.47
α-phellandrene	KI,W	997	1001	-	9.26	0.11
Trans β-ocymene	KI,W	1001	1003	0.09	-	-
Hexyl acetate	KI,W	1008	1008	0.65	-	-
α-terpinene	KI,W	1009	1018	-	-	0.05
Para cymene	KI,W	1020	1026	0.14	2.48	0.24
1,8-cineole	KI,W	1027	1033	1.71	-	-
Limonene	KI,W	1031	1036	-	26.44	4.74
Cis β-ocimene	KI,W	1038	1040	-	1.60	-
Trans β-ocymene	KI,W	1043	1045	5.05	0.50	-
γ-terpinene	KI,W	1055	1059	-	0.34	0.31
Trans linalool oxide	KI,W	1063	1065	3.44	-	-
Cis linalool oxide	KI,W	1067	1069	0.12	-	-
Terpinolene	KI,W	1080	1084	0.07	-	0.07
Para cymenyl	KI	1084		-	-	0.21
Fenchone	KI,W	1090	1094	-	21.68	-
Linalool	KI,W	1105	1100	34.44	-	-
3-octyl acetate	KI,W	1119	1122	0.12	-	-
Neo-allo-ocymene	KI,W	1125	1131	0.23	-	-
Camphor	KI,W	1137	1139	0.31	0.43	-
1-terpinen-4-ol	KI,W	1177	1179	2.43	0.18	-
Hexyl butyrate	KI,W	1187	1191	0.41	-	-
Myrtenal	KI,W	1188	1193	-	-	0.17
Methyl chavicol	KI,W	1194	1196	-	2.19	-
α-terpineol	KI,W	1198	1195	1	-	-
Trans-Carveol	KI,W	1221	1225	0.19	-	-
Linalyl acetate	KI,W	1254	1261	34.19	-	-
Phellandral	KI,W	1270	1275	-	-	0.10
Bornyl acetate	KI,W	1279	1285	0.19	-	-
Lavandulyl acetate	KI,W	1284	1289	4.08	-	-
Anethole	KI,W	1288	1289	-	23.50	-
Neryl acetate	KI,W	1356	1362	0.33	-	-
Geranyl acetate	KI,W	1376	1383	0.49	-	-
Hexyl caproate	KI,W	1381	1380	0.10	-	-
β-caryophyllene	KI,W	1424	1428	3.83	-	0.07
α-farnesene	KI,W	1429	1433	0.09	-	-
β-farnesene	KI,W	1453	1458	3.08	-	0.19
Germacrene D	KI,W	1478	1480	0.39	-	0.05
α-amorphene	KI,W	1509	1506	0.07	-	-
Myristicin	KI,W	1528	1526	-	-	36.15
Elemicin	KI,W	1550	1554	-	-	2.74
Caryophyllene oxide	KI,W	1578	1573	0.21	-	-
Allyl tetramethoxybenzene	KI,W	1590	1591	-	-	6.45
Apiole	KI,W	1684	1685	-	-	20.97

^1^ “KI, W” means that identification was based on Kováts indexes and comparison with Wiley library. “W” means that identification was based on comparison with Wiley library; ^2^ NIST database [27].

**Table 2:** Total phenolic content (mg of Gallic Acid Equivalents/L) in organic fennel, parsley, and lavender essential oils from Spain.

Essential Oil	Total Phenols ^¥^ GAE/(mg/L)
*Foeniculum vulgare*	262.59 ^c^ ± 15.5
*Petroselinum crispum*	388.35 ^d^ ± 21.7
*Lavandula officinalis*	137.52 ^b^ ± 38.3

^¥^ Gallic Acid Equivalent (mg/L); Values followed by the same small letter within the same column are not significantly different (*p* > 0.05) according to Tukey’s Multiple Range Test.

**Table 3:** Antioxidant activity of organic fennel, parsley, and lavender essential oils from Spain using the corresponding concentrations (A = 5 g/L, B = 10 g/L, C = 20 g/L, D = 50 g/L) measured by DPPH method.

	DPPH % Inhibition				
	A	B	C	D	IC_50_
*Foeniculum vulgare*	11.24 ^aA^ ± 0.80	14.99 ^bA^ ± 1.12	21.88 ^cA^ ± 4.25	21.17 ^cA^ ± 2.20	45.89 *
*Petroselinum crispum*	28.87 ^aB^ ± 0.61	44.22 ^bC^ ± 0.55	53.34 ^cC^ ± 1.87	64.28 ^dC^ ± 1.45	12.91 *
*Lavandula officinalis*	13.07 ^aA^ ± 1.15	19.60 ^bB^ ± 1.00	28.32 ^cB^ ± 2.55	33.54 ^dB^ ± 1.03	31.30 *
BHT	95.4 ^aC^ ± 0.28	96.33 ^aD^ ± 1.02	96.98 ^aD^ ± 0.51	97.43 ^aD^ ± 0.93	0.53 *

^¥^ IC_50_: Concentration (g/L) for a 50% inhibition; Values followed by the same small letter within the same line are not significantly different (*p* > 0.05) according to Tukey’s Multiple Range Test; Values followed by the same capital letter within the same column are not significantly different (*p* > 0.05) according to Tukey’s Multiple Range Test.

**Table 4:** Antioxidant activity of fennel, parsley, and lavender essential oils using the corresponding concentrations (A = 5 g/L, B = 10 g/L, C = 20 g/L, D = 50 g/L) measured by FRAP method.

	FRAP			
	TEAC ^¥^ (mmol/L Trolox)			
	A	B	C	D
*Foeniculum vulgare*	0.19 ^aB^ ± 0.01	0.26 ^bB^ ± 0.03	0.31 ^cB^ ± 0.01	0.37 ^dB^ ± 0.02
*Petroselinum crispum*	0.40 ^aC^ ± 0.00	0.56 ^bC^ ± 0.07	0.82 ^cC^ ± 0.06	0.93 ^dC^ ± 0.07
*Lavandula officinalis*	0.14 ^aA^ ± 0.02	0.18 ^bA^ ± 0.02	0.23 ^cA^ ± 0.02	0.24 ^cA^ ± 0.01
BHT	2.08 ^aD^ ± 0.05	2.57 ^bD^ ± 0.09	2.94 ^cD^ ± 0.06	3.39 ^dD^ ± 0.05

^¥^ (TEAC): Trolox equivalent antioxidant capacity; Values followed by the same small letter within the same line are not significantly different (*p* > 0.05) according to Tukey’s Multiple Range Test; Values followed by the same capital letter within the same column are not significantly different (*p* > 0.05) according to Tukey’s Multiple Range Test.

**Table 5:** Antioxidant activity of organic fennel, parsley, and lavender essential oils from Spain using the corresponding concentrations (A = 5 g/L, B = 10 g/L, C = 20 g/L, D = 50 g/L) measured by TBARS assay.

	TBARS Inhibition %			
	A	B	C	D
*Foeniculum vulgare*	51.06 ^aB^ ± 1.04	53.24 ^aA^ ± 1.98	51.46 ^aA^ ± 1.37	52.35 ^aA^ ± 1.50
*Petroselinum crispum*	12.33 ^A^ ± 1.68	-	-	-
*Lavandula officinalis*	61.52 ^aC^ ± 0.21	60.38 ^aB^ ± 2.42	57.50 ^aB^ ± 1.19	56.91 ^aA^ ± 3.15
BHT	84.71 ^aC^ ± 0.06	87.03 ^bC^ ± 0.10	88.97 ^bC^ ± 0.25	90.22 ^bB^ ± 0.02

Values followed by the same small letter within the same line are not significantly different (*p* > 0.05) according to Tukey’s Multiple Range Test; Values followed by the same capital letter within the same column are not significantly different (*p* > 0.05) according to Tukey’s Multiple Range Test.

**Table 6:** Summary of antioxidant ability of organic fennel, parsley, and lavender essential oils from Spain as assessed by three antioxidant methods and TPC.

Method	*Foeniculum vulgare*	*Petroselium crispum*	*Lavandula officinalis*
DPPH	(*)	(***)	(**)
FRAP	(*)	(**)	(*)
TBARS	(***)	(--)	(***)
TPC ^1^	(***)	(****)	(**)

(--) Pro-oxidant; (*) low: less than 25% of the antioxidant effect of reference BHT; (**) low-medium: between 26% and 50% of the antioxidant effect of reference BHT; (***) medium-high: between 51% and 75% of the activity of reference BHT; (****) high antioxidant activity: >76% of the activity of reference BHT; ^1^ (TPC) increased numbers of asterisks indicate increased total phenolic content.

**Table 7:** The volume effect (VE) of organic fennel, parsley, and lavender essential oils from Spain on the inhibition of *Listeria innocua* CECT910 and *Pseudomonas fluorescens* CECT 844.

Essential Oil	Volume * μL	^¥^ Diameter of Inhibition Zone (mm) Including Disc Diameter of 9 mm	
		*Listeria innocua*	*Pseudomonas fluorescens*
*Foeniculum vulgare*	40	10.75 ^b^ ± 0.35	N.A.
	20	9.55 ^a^ ± 0.17	N.A.
	10	N.A.	N.A.
	5	N.A.	N.A.
	2	N.A.	N.A.
*Petroselinum crispum*	40	11.42 ^b^ ± 0.11	N.A.
	20	11.16 ^b^ ± 0.05	N.A.
	10	10.25 ^b^ ± 0.00	N.A.
	5	10.20 ^b^ ± 0.35	N.A.
	2	N.A.	N.A.
*Lavandula officinalis*	40	17.00 ^d^ ± 0.00	N.A.
	20	13.25 ^c^ ± 1.77	N.A.
	10	N.A.	N.A.
	5	N.A.	N.A.
	2	N.A.	N.A.

^¥^ (mean and SD); * Volumes of essential oil in the discs; ^a–e^ For the same essential oil, values followed by different letters within the same column are significantly different (*p* < 0.05) according to Tukey’s multiple range test; N.A. non-active.
